# Supplementary material for: Stable Protein Sialylation in Physcomitrella
Source: Front Plant Sci. 2020 Dec 18;11:610032. doi: 10.3389/fpls.2020.610032 (PMC7775405; doi:10.3389/fpls.2020.610032)
Supplement: Supplementary file 1 [file Data_Sheet_1.docx]

**Frontiers in Plant Science**

**doi: 10.3389/fpls.2020.610032**

**Supplementary Material**

Stable Protein Sialylation in Physcomitrella

Lennard L. Bohlender^1^, Juliana Parsons^1^, Sebastian N. W. Hoernstein^1^, Christine Rempfer^1, 2^, Natalia Ruiz-Molina^1^, Timo Lorenz^1^, Fernando Rodríguez Jahnke^1,2^, Rudolf Figl^3^, Benjamin Fode^4^, Friedrich Altmann^3^, Ralf Reski^1, 2, 5, 6^ and Eva L. Decker^1*^

^1^Plant Biotechnology, Faculty of Biology, University of Freiburg, Freiburg, Germany

^2^Spemann Graduate School of Biology and Medicine (SGBM), University of Freiburg, Freiburg, Germany

^3^Deparment of Chemistry, University of Natural Resources and Life Sciences, Vienna, Austria

^4^Eleva GmbH, Freiburg, Germany

^5^Signalling Research Centres BIOSS and CIBSS, University of Freiburg, Freiburg, Germany

^6^Cluster of Excellence livMatS @ FIT – Freiburg Center for Interactive Materials and Bioinspired Technologies, University of Freiburg, Freiburg, Germany

***Correspondence:**Eva L. Decker
[eva.decker@biologie.uni-freiburg.de](javascript:linkTo_UnCryptMailto('ocknvq,gxc0fgemgtBdkqnqikg0wpk//htgkdwti0fg');)

**Supplementary Table 1.** Primers used. Introduced restriction sites are written in italic. Lower-case letters indicate sequences of primer overhangs. Bolt letters indicate sequence mismatches for site-directed mutagenesis.

| 1 | GNE_BamHI_fwd | TTT*GGATCC*ATGGAGAAGAATGGAAATAACCGA |
| --- | --- | --- |
| 2 | GNE_SalI_rev | TTT*GTCGAC*CTAGTAGATCCTGCGTGTTGT |
| 3 | NANS_BamHI_fwd | TTT*GGATCC*ATGCCGCTGGAGCTG |
| 4 | NANS_SalI_rev | TTT*GTCGAC*TTAAGACTTGATTTTTTTGCCATGATTATCTAC |
| 5 | NANP_BamHI_fwd | TTT*GGATCC*ATGGGGCTGAGCCGC |
| 6 | NANP_SalI_rev | TTT*GTCGAC*TTAAGTGGACATACTGACTTTGCAGTCTA |
| 7 | CMAS_trc_XhoI_fwd | TTT*CTCGAG*ATGAAGCCCCCGCA |
| 8 | CMAS_KpnI_rev | TTT*GGTACC*CTATTTTTGGCATGAATTATTAACCTTTTCC |
| 9 | CSAT_BamHI_fwd | TTT*GGATCC*ATGGCTGCCCCGAGA |
| 10 | CSAT_SalI_rev | TTT*GTCGAC*TCACACACCAATAACTCTCTCCTTT |
| 11 | ST6Gal1_BamHI_fwd | TTT*GGATCC*ATGATTCACACCAACCTGAAGAAA |
| 12 | ST6Gal1_SalI_rev | TTT*GTCGAC*TTAGCAGTGAATGGTCCGG |
| 13 | BSD_35S_P_AgeI_fwd | TTT*ACCGGT*CTACTCCAAAAATGTCAAAGATACAG |
| 14 | BSD_35S_T_AatII_rev | AGTTT*GACGTC*GGATTTTAGTACTGGATTTTGGTT |
| 15 | GNE_AgeI_35S_P_fwd | TTT*ACCGGT*ATGCCTGCAGGTCCC |
| 16 | GNE_NosT_SgrDI_rev | TTTT*CGTCGACG*CGATCTAGTAACATAGATGACACCG |
| 17 | NANS/CMAS_SgrDI_35S_P_fwd | TTTT*CGTCGACG*ATGCCTGCAGGTCCC |
| 18 | NANS/CSAT_NosT_AvrII_rev | TTT*CCTAGG*CGATCTAGTAACATAGATGACACCG |
| 19 | NANP/ST_AvrII_35S_fwd | TTT*CCTAGG*ATGCCTGCAGGTCCC |
| 20 | NANP/CMAS_NosT_AscI_rev | TTT*GGCGCGCC*CGATCTAGTAACATAGATGACACCG |
| 21 | CSAT_AscI_35S_P_fwd | TTT*GGCGCGCC*ATGCCTGCAGGTCCC |
| 22 | ST_NosT_AgeI_rev | TTT*ACCGGT*CGATCTAGTAACATAGATGACACCG |
| 23 | APT_5HR_MCS_fwd | TTT*GCTCTTC*GAAAGCTTATGCCAATAGAAAAGATCA |
| 24 | APT_5HR_MCS_rev | AAA*CCTAGG*AAAA*CGTCGAC*GTTT*ACCGGT*AAATTAATCCAGTCTGTGTCAAGT |
| 25 | APT_3HR_MCS_fwd | TT*CCTAGG*TTT*GGCGCGCC*TGGTACCGATTGCATTGAGATG |
| 26 | APT_3HR_MCS_rev | AAA*GCTCTTC*TAAAAGCTTACTCGCCCTCATATTC |
| 27 | P4H1_5HR_NotI_fwd | TTT*GCGGCCGC*TACACCATAGAAGGTAGCAAC |
| 28 | P4H1_5HR_MCS_rev | AA*CCTAGG*TTT*GGCGCGCC*AAAA*CGTCGAC*GTTCGAACCTTACTCTCGATTC |
| 29 | P4H1_3HR_MCS_fwd | TT*CCTAGG*TTT*ACCGGT*AGCTA*GACGTC*AAGCTTGAGCTCCTCCAAGTT |
| 30 | P4H1_3HR_NotI_rev | GTT*GCGGCCGC*GAATTCAGAGGGTAGGATTGTGTG |
| 31 | SDM_GNEmut_fwd | ATGC**AG**AAGAAGGGCATCGAGCATC |
| 32 | SDM_GNEmut_rev | CAC**TA**GAACCATCTCCTTGCTGCC |
| 33 | XhoI_1,4FT5'UTR_fwd | *CTCGAG*GGTCTCCTGCTCACAAACAGTC |
| 34 | FT_CTS_GT4_J_R | gcgcggctgggaggaggcCACTGTGGGGTTTTGAAGACTGCGT |
| 35 | GT4_FT_CTS_J_F | ttcaaaaccccacagtgGCCTCCTCCCAGCCGCGC |
| 36 | GT4_J_R | GCTCGGTGTCCCGATGTCCAC |
| 37 | GT4_P2A_1_J_R | ccagcctgtttcagaaggctgaagttggtagcGCTCGGTGTCCCGATGTCCACTG |
| 38 | GT4_P2A_2_J_R | t*ctcgag*agggccagggttctcttcaacatctCCAGCCTGTTTCAGAAGGCTGAAGTTGG |
| 39 | BamHI_35S_long_fwd | *GGATCC*AAGCTTGCATGCCTGC |
| 40 | XhoI_35S_long_rev | *CTCGAG*TTATATAGAGGAAGGGTCTTGCG |
| 41 | P4H2-5´-fwd | ccgccagatcttccggatggctcgagtttttcagcaagatatcGCTCAGTTTCGTACGAGCGATTTG |
| 42 | P4H2-5´-rev | ccctgcgccatcagatccttggcggAACCCACGCTCAGCAGCACA |
| 43 | P4H2-3´-fwd | cggtgtcatctatgttactagatcgCTGCTCTTCTTCAGCCTACA |
| 44 | P4H2-3´-rev | atggcagctgagaatattgtaggagatcttctagaaagatatcGTCTTCGTTAATTAATCCACTTG |
| 45 | P4H2-hpt-fwd | gatcctgtgctgctgagcgtgggtgggttgttCCGCCAAGGATCTGATG |
| 46 | P4H2-hpt-rev | aggggtgtaggctgaagaagagcagCGATCTAGTAACATAGATGACACC |
| 47 | P4H2_35Slong_FTCTS_fw | ggaacgccacggtttcacattggttacacgAGATTAGCCTTTTCAATTTCAGAAAG |
| 48 | P4H2_35Slong_FTCTS_rev | tgagaacacaacctgggaagcagaccccatCACTGTGGGGTTTTGAAGAC |
| 49 | P4H2_rST_nosT_fw | actttacgcagtcttcaaaaccccacagtgATGGGGTCTGCTTCCCAG |
| 50 | P4H2_rST_nosT_rev | cgaaagcggagacagtgaggagcctgagacCGATCTAGTAACATAGATGACACCGC |
| 51 | EF1a_fw | AGCGTGGTATCACAATTGAC |
| 52 | EF1a_rev | GATCGCTCGATCATGTTATC |
| 53 | Leaf_APT_HR5_fwd | GCCTTTTGAACAAGATGACAAAGTG |
| 54 | Leaf_GNE_mus_rev | AACAATCGTGTGGAGCCTGG |
| 55 | Leaf_NANP_fwd | CTGGGGACTGTGTGATGGTC |
| 56 | Leaf_APT_HR3_rev | GCAGTACGATCTGCTTTTGCC |
| 57 | P4Hsc8KO_1547F | GGCTAATGATGAAGATGCGAGA |
| 58 | CMAS_leaf_rev | GGAAGGCCCCTGAATCCA |
| 59 | full length mrna BSD Stop | GTTATGTGTGGGAGGGCTAA |
| 60 | P4Hsc8KO_4023r | AGCATCCCCTCGTTTAGGTT |
| 61 | RT1 | TGTCGTGCTCCACCATGTTG |
| 62 | B1,3galT3_RT_879F | ACTTGGACAGTAAGCAGAGATTGG |
| 63 | 3HR_zeo_fwd | CTTCGTGGCCGAGGAGCAGG |
| 64 | 3genome_GalT3_rev | CGAAACTTACCGGAGCCGGCG |
| 65 | Leaf_P4H2_5HR_f | GGCATTTGTGCCGTTTATGC |
| 66 | Leaf_P4H2_5HR_rev | GGAAGGAAGGCTGTGGTTTG |
| 67 | qhpt_f | ATACGAGGTCGCCAACATCT |
| 68 | P4H2_3HR_rev | CACAAACTTCATCGACTTGATCC |
| 69 | EF1a_qf | CGACGCCCCTGGACATC |
| 70 | EF1a_qr | CCTGCGAGGTTCCCGTAA |
| 71 | c45-tg2F | ACGCACCGGCATCGT |
| 72 | c45-tg2R | TGCTTGTTCATCACGACACCA |
| 73 | GNE_mus_qPCR_fwd | CTACCGGACGTCCTCAATCG |
| 74 | GNE_mus_qPCR_rev | CGTCAAATCGGTCTCCGTGA |
| 75 | NANS_qPCR_fwd | TTGCAGTGTACCAGCGCATA |
| 76 | NANS_qPCR_rev | TATTCCGAGATGACCCGCAG |
| 77 | NANP_qPCR_fwd | GCTTGTGCCTGTCAGTCCTA |
| 78 | NANP_qPCR_rev | ATGGACGGTGCTGGTTTCTC |
| 79 | qPCR CMAS fwd | CTTGAAGAGAGTGGGCCTAAGT |
| 80 | qPCR CMAS rev | GCCTTCTGGGCAGTAGAACA |
| 81 | CSAT_mus_qPCR_fwd | TTCCGACACTTCCCTTTGGG |
| 82 | CSAT_mus_qPCR_rev | ACGTCACAACGATCCCTGAC |
| 83 | ST6_rat_qPCR_fwd | AAACTCCCAGCTTGGTCGAG |
| 84 | ST6_rat_qPCR_rev | GGTAGGGGCCCCATTAAACC |
| 85 | qPCR chGalT4 fw | TTGACCTCGGTCCCAGTG |
| 86 | qPCR chGalT4 rev | GGCATGTTAAACTCAATCAGCA |

**Supplementary Table 2.** Monoisotopic masses of glycopeptides used for mass spectrometric analysis. All combinations of the searched peptides with possible glycan structures are given in three calculated charge stages ([M+nH]^n+^). cam: carbamidomethylation (C, + 57.021464 Da, fixed); ox: oxidation (M, +15.994915 Da, optional); M: mannose, Gn: *N*-acetylglucosamine, A: galactose, Na: sialic acid, P: pentose.

| **Glycopeptide** | **[M+2H]^2+^** | **[M+3H]^3+^** | **[M+4H]^4+^** |
| --- | --- | --- | --- |
| IPCcamSQPPQIEHGTINSSR_AA | 1822.2879 | 1215.1944 | 911.6476 |
| IPCcamSQPPQIEHGTINSSR_AA_2P | 1954.3302 | 1303.2226 | 977.6688 |
| IPCcamSQPPQIEHGTINSSR_AA_3P | 2020.3514 | 1347.2367 | 1010.6793 |
| IPCcamSQPPQIEHGTINSSR_AA_4P | 2086.3725 | 1391.2508 | 1043.6899 |
| IPCcamSQPPQIEHGTINSSR_AA_5P | 2152.3936 | 1435.2649 | 1076.7005 |
| IPCcamSQPPQIEHGTINSSR_AA_6P | 2218.4148 | 1479.2790 | 1109.7110 |
| IPCcamSQPPQIEHGTINSSR_AA_P | 1888.3091 | 1259.2085 | 944.6582 |
| IPCcamSQPPQIEHGTINSSR_AGn | 1741.2615 | 1161.1768 | 871.1344 |
| IPCcamSQPPQIEHGTINSSR_AGn_2P | 1873.3038 | 1249.2050 | 937.1556 |
| IPCcamSQPPQIEHGTINSSR_AGn_3P | 1939.3250 | 1293.2191 | 970.1661 |
| IPCcamSQPPQIEHGTINSSR_AGn_4P | 2005.3461 | 1337.2332 | 1003.1767 |
| IPCcamSQPPQIEHGTINSSR_AGn_5P | 2071.3673 | 1381.2473 | 1036.1873 |
| IPCcamSQPPQIEHGTINSSR_AGn_6P | 2137.3884 | 1425.2614 | 1069.1979 |
| IPCcamSQPPQIEHGTINSSR_AGn_P | 1807.2827 | 1205.1909 | 904.1450 |
| IPCcamSQPPQIEHGTINSSR_AM | 1639.7218 | 1093.4836 | 820.3646 |
| IPCcamSQPPQIEHGTINSSR_AM_2P | 1771.7641 | 1181.5118 | 886.3857 |
| IPCcamSQPPQIEHGTINSSR_AM_3P | 1837.7853 | 1225.5259 | 919.3963 |
| IPCcamSQPPQIEHGTINSSR_AM_4P | 1903.8064 | 1269.5400 | 952.4069 |
| IPCcamSQPPQIEHGTINSSR_AM_5P | 1969.8276 | 1313.5541 | 985.4174 |
| IPCcamSQPPQIEHGTINSSR_AM_6P | 2035.8487 | 1357.5682 | 1018.4280 |
| IPCcamSQPPQIEHGTINSSR_AM_P | 1705.7430 | 1137.4977 | 853.3751 |
| IPCcamSQPPQIEHGTINSSR_GnGn | 1660.2351 | 1107.1592 | 830.6212 |
| IPCcamSQPPQIEHGTINSSR_GnM | 1558.6954 | 1039.4660 | 779.8514 |
| IPCcamSQPPQIEHGTINSSR_GnM5 | 1720.7482 | 1147.5012 | 860.8777 |
| IPCcamSQPPQIEHGTINSSR_M4 | 1538.1821 | 1025.7905 | 769.5947 |
| IPCcamSQPPQIEHGTINSSR_M5 | 1619.2085 | 1079.8081 | 810.1079 |
| IPCcamSQPPQIEHGTINSSR_M6 | 1700.2349 | 1133.8257 | 850.6211 |
| IPCcamSQPPQIEHGTINSSR_M7 | 1781.2613 | 1187.8433 | 891.1343 |
| IPCcamSQPPQIEHGTINSSR_M8 | 1862.2877 | 1241.8609 | 931.6475 |
| IPCcamSQPPQIEHGTINSSR_M9 | 1943.3141 | 1295.8785 | 972.1607 |
| IPCcamSQPPQIEHGTINSSR_MM | 1457.1557 | 971.7729 | 729.0815 |
| IPCcamSQPPQIEHGTINSSR_NaA_2P | 2033.8568 | 1356.2403 | 1017.4320 |
| IPCcamSQPPQIEHGTINSSR_NaA_3P | 2099.8779 | 1400.2544 | 1050.4426 |
| IPCcamSQPPQIEHGTINSSR_NaA_4P | 2165.8990 | 1444.2685 | 1083.4532 |
| IPCcamSQPPQIEHGTINSSR_NaA_5P | 2231.9202 | 1488.2826 | 1116.4637 |
| IPCcamSQPPQIEHGTINSSR_NaA_6P | 2297.9414 | 1532.2967 | 1149.4743 |
| IPCcamSQPPQIEHGTINSSR_NaA_P | 1967.8356 | 1312.2262 | 984.4215 |
| IPCcamSQPPQIEHGTINSSR_NaGn | 1886.8092 | 1258.2086 | 943.9083 |
| IPCcamSQPPQIEHGTINSSR_NaGn_P | 1952.8303 | 1302.2227 | 976.9188 |
| IPCcamSQPPQIEHGTINSSR_NaM | 1785.2695 | 1190.5154 | 893.1384 |
| IPCcamSQPPQIEHGTINSSR_NaNa | 2113.3833 | 1409.2580 | 1057.1953 |
| IPCcamSQPPQIEHGTINSSR_NaNa_P | 2245.4256 | 1497.2861 | 1123.2164 |
| ISEENETTCcamYMGK_AA | 1592.6199 | 1062.0824 | 796.8136 |
| ISEENETTCcamYMGK_AA_2P | 1724.6622 | 1150.1106 | 862.8348 |
| ISEENETTCcamYMGK_AA_3P | 1790.6834 | 1194.1247 | 895.8453 |
| ISEENETTCcamYMGK_AA_4P | 1856.7045 | 1238.1388 | 928.8559 |
| ISEENETTCcamYMGK_AA_5P | 1922.7257 | 1282.1529 | 961.8665 |
| ISEENETTCcamYMGK_AA_6P | 1988.7468 | 1326.1670 | 994.8771 |
| ISEENETTCcamYMGK_AA_P | 1658.6411 | 1106.0965 | 829.8242 |
| ISEENETTCcamYMGK_AGn | 1511.5935 | 1008.0648 | 756.3004 |
| ISEENETTCcamYMGK_AGn_2P | 1643.6358 | 1096.0930 | 822.3216 |
| ISEENETTCcamYMGK_AGn_3P | 1709.6570 | 1140.1071 | 855.3321 |
| ISEENETTCcamYMGK_AGn_4P | 1775.6781 | 1184.1212 | 888.3427 |
| ISEENETTCcamYMGK_AGn_5P | 1841.6993 | 1228.1353 | 921.3533 |
| ISEENETTCcamYMGK_AGn_6P | 1907.7204 | 1272.1494 | 954.3639 |
| ISEENETTCcamYMGK_AGn_P | 1577.6146 | 1052.0789 | 789.3110 |
| ISEENETTCcamYMGK_AM | 1410.0538 | 940.3716 | 705.5306 |
| ISEENETTCcamYMGK_AM_2P | 1542.0961 | 1028.3998 | 771.5517 |
| ISEENETTCcamYMGK_AM_3P | 1608.1173 | 1072.4139 | 804.5623 |
| ISEENETTCcamYMGK_AM_4P | 1674.1384 | 1116.4280 | 837.5729 |
| ISEENETTCcamYMGK_AM_5P | 1740.1596 | 1160.4421 | 870.5834 |
| ISEENETTCcamYMGK_AM_6P | 1806.1807 | 1204.4562 | 903.5940 |
| ISEENETTCcamYMGK_AM_P | 1476.0750 | 984.3857 | 738.5411 |
| ISEENETTCcamYMGK_GnGn | 1430.5671 | 954.0472 | 715.7872 |
| ISEENETTCcamYMGK_GnM | 1329.0274 | 886.3540 | 665.0174 |
| ISEENETTCcamYMGK_GnM5 | 1491.0802 | 994.3892 | 746.0438 |
| ISEENETTCcamYMGK_M4 | 1308.5141 | 872.6785 | 654.7607 |
| ISEENETTCcamYMGK_M5 | 1389.5405 | 926.6961 | 695.2739 |
| ISEENETTCcamYMGK_M6 | 1470.5669 | 980.7137 | 735.7871 |
| ISEENETTCcamYMGK_M7 | 1551.5933 | 1034.7313 | 776.3003 |
| ISEENETTCcamYMGK_M8 | 1632.6197 | 1088.7489 | 816.8135 |
| ISEENETTCcamYMGK_M9 | 1713.6461 | 1142.7665 | 857.3267 |
| ISEENETTCcamYMGK_MM | 1227.4877 | 818.6609 | 614.2475 |
| ISEENETTCcamYMGK_NaA_2P | 1804.1888 | 1203.1283 | 902.5980 |
| ISEENETTCcamYMGK_NaA_3P | 1870.2099 | 1247.1424 | 935.6086 |
| ISEENETTCcamYMGK_NaA_4P | 1936.2311 | 1291.1565 | 968.6192 |
| ISEENETTCcamYMGK_NaA_5P | 2002.2522 | 1335.1706 | 1001.6298 |
| ISEENETTCcamYMGK_NaA_6P | 2068.2733 | 1379.1847 | 1034.6403 |
| ISEENETTCcamYMGK_NaA_P | 1738.1676 | 1159.1142 | 869.5875 |
| ISEENETTCcamYMGK_NaGn | 1657.1412 | 1105.0966 | 829.0743 |
| ISEENETTCcamYMGK_NaGn_P | 1723.1623 | 1149.1107 | 862.0848 |
| ISEENETTCcamYMGK_NaM | 1555.6015 | 1037.4034 | 778.3044 |
| ISEENETTCcamYMGK_NaNa | 1883.7153 | 1256.1460 | 942.3613 |
| ISEENETTCcamYMGK_NaNa_P | 2015.7576 | 1344.1741 | 1008.3824 |
| ISEENETTCcamYMoxGK_AA | 1600.6174 | 1067.4140 | 800.8123 |
| ISEENETTCcamYMoxGK_AA_2P | 1732.6597 | 1155.4422 | 866.8335 |
| ISEENETTCcamYMoxGK_AA_3P | 1798.6808 | 1199.4563 | 899.8441 |
| ISEENETTCcamYMoxGK_AA_4P | 1864.7020 | 1243.4704 | 932.8546 |
| ISEENETTCcamYMoxGK_AA_5P | 1930.7231 | 1287.4845 | 965.8652 |
| ISEENETTCcamYMoxGK_AA_6P | 1996.7443 | 1331.4986 | 998.8758 |
| ISEENETTCcamYMoxGK_AA_P | 1666.6385 | 1111.4281 | 833.8229 |
| ISEENETTCcamYMoxGK_AGn | 1519.5910 | 1013.3964 | 760.2991 |
| ISEENETTCcamYMoxGK_AGn_2P | 1651.6333 | 1101.4246 | 826.3203 |
| ISEENETTCcamYMoxGK_AGn_3P | 1717.6544 | 1145.4387 | 859.3309 |
| ISEENETTCcamYMoxGK_AGn_4P | 1783.6756 | 1189.4528 | 892.3414 |
| ISEENETTCcamYMoxGK_AGn_5P | 1849.6967 | 1233.4669 | 925.3520 |
| ISEENETTCcamYMoxGK_AGn_6P | 1915.7179 | 1277.4810 | 958.3626 |
| ISEENETTCcamYMoxGK_AGn_P | 1585.6121 | 1057.4105 | 793.3097 |
| ISEENETTCcamYMoxGK_AM | 1418.0513 | 945.7033 | 709.5293 |
| ISEENETTCcamYMoxGK_AM_2P | 1550.0936 | 1033.7315 | 775.5504 |
| ISEENETTCcamYMoxGK_AM_3P | 1616.1147 | 1077.7456 | 808.5610 |
| ISEENETTCcamYMoxGK_AM_4P | 1682.1359 | 1121.7597 | 841.5716 |
| ISEENETTCcamYMoxGK_AM_5P | 1748.1570 | 1165.7738 | 874.5822 |
| ISEENETTCcamYMoxGK_AM_6P | 1814.1782 | 1209.7879 | 907.5927 |
| ISEENETTCcamYMoxGK_AM_P | 1484.0724 | 989.7174 | 742.5399 |
| ISEENETTCcamYMoxGK_GnGn | 1438.5646 | 959.3788 | 719.7859 |
| ISEENETTCcamYMoxGK_GnM | 1337.0249 | 891.6857 | 669.0161 |
| ISEENETTCcamYMoxGK_GnM5 | 1499.0777 | 999.7209 | 750.0425 |
| ISEENETTCcamYMoxGK_M4 | 1316.5116 | 878.0101 | 658.7594 |
| ISEENETTCcamYMoxGK_M5 | 1397.5380 | 932.0277 | 699.2726 |
| ISEENETTCcamYMoxGK_M6 | 1478.5644 | 986.0453 | 739.7858 |
| ISEENETTCcamYMoxGK_M7 | 1559.5908 | 1040.0629 | 780.2990 |
| ISEENETTCcamYMoxGK_M8 | 1640.6172 | 1094.0805 | 820.8122 |
| ISEENETTCcamYMoxGK_M9 | 1721.6436 | 1148.0981 | 861.3254 |
| ISEENETTCcamYMoxGK_MM | 1235.4852 | 823.9925 | 618.2462 |
| ISEENETTCcamYMoxGK_NaA_2P | 1812.1862 | 1208.4599 | 906.5968 |
| ISEENETTCcamYMoxGK_NaA_3P | 1878.2074 | 1252.4740 | 939.6073 |
| ISEENETTCcamYMoxGK_NaA_4P | 1944.2285 | 1296.4881 | 972.6179 |
| ISEENETTCcamYMoxGK_NaA_5P | 2010.2497 | 1340.5022 | 1005.6285 |
| ISEENETTCcamYMoxGK_NaA_6P | 2076.2708 | 1384.5163 | 1038.6391 |
| ISEENETTCcamYMoxGK_NaA_P | 1746.1651 | 1164.4458 | 873.5862 |
| ISEENETTCcamYMoxGK_NaGn | 1665.1387 | 1110.4282 | 833.0730 |
| ISEENETTCcamYMoxGK_NaGn_P | 1731.1598 | 1154.4423 | 866.0835 |
| ISEENETTCcamYMoxGK_NaM | 1563.5990 | 1042.7351 | 782.3031 |
| ISEENETTCcamYMoxGK_NaNa | 1891.7128 | 1261.4776 | 946.3600 |
| ISEENETTCcamYMoxGK_NaNa_P | 2023.7550 | 1349.5058 | 1012.3812 |
| MDGASNVTCcamINSR_AA | 1524.1073 | 1016.4073 | 762.5573 |
| MDGASNVTCcamINSR_AA_2P | 1656.1496 | 1104.4355 | 828.5785 |
| MDGASNVTCcamINSR_AA_3P | 1722.1708 | 1148.4496 | 861.5890 |
| MDGASNVTCcamINSR_AA_4P | 1788.1919 | 1192.4637 | 894.5996 |
| MDGASNVTCcamINSR_AA_5P | 1854.2131 | 1236.4778 | 927.6102 |
| MDGASNVTCcamINSR_AA_6P | 1920.2342 | 1280.4919 | 960.6208 |
| MDGASNVTCcamINSR_AA_P | 1590.1285 | 1060.4214 | 795.5679 |
| MDGASNVTCcamINSR_AGn | 1443.0809 | 962.3897 | 722.0441 |
| MDGASNVTCcamINSR_AGn_2P | 1575.1232 | 1050.4179 | 788.0652 |
| MDGASNVTCcamINSR_AGn_3P | 1641.1444 | 1094.4320 | 821.0758 |
| MDGASNVTCcamINSR_AGn_4P | 1707.1655 | 1138.4461 | 854.0864 |
| MDGASNVTCcamINSR_AGn_5P | 1773.1867 | 1182.4602 | 887.0970 |
| MDGASNVTCcamINSR_AGn_6P | 1839.2078 | 1226.4743 | 920.1076 |
| MDGASNVTCcamINSR_AGn_P | 1509.1020 | 1006.4038 | 755.0547 |
| MDGASNVTCcamINSR_AM | 1341.5412 | 894.6966 | 671.2743 |
| MDGASNVTCcamINSR_AM_2P | 1473.5835 | 982.7248 | 737.2954 |
| MDGASNVTCcamINSR_AM_3P | 1539.6047 | 1026.7389 | 770.3060 |
| MDGASNVTCcamINSR_AM_4P | 1605.6258 | 1070.7530 | 803.3166 |
| MDGASNVTCcamINSR_AM_5P | 1671.6470 | 1114.7671 | 836.3271 |
| MDGASNVTCcamINSR_AM_6P | 1737.6681 | 1158.7812 | 869.3377 |
| MDGASNVTCcamINSR_AM_P | 1407.5624 | 938.7107 | 704.2848 |
| MDGASNVTCcamINSR_GnGn | 1362.0545 | 908.3721 | 681.5309 |
| MDGASNVTCcamINSR_GnM | 1260.5148 | 840.6790 | 630.7611 |
| MDGASNVTCcamINSR_GnM5 | 1422.5676 | 948.7142 | 711.7875 |
| MDGASNVTCcamINSR_M4 | 1240.0015 | 827.0034 | 620.5044 |
| MDGASNVTCcamINSR_M5 | 1321.0279 | 881.0210 | 661.0176 |
| MDGASNVTCcamINSR_M6 | 1402.0543 | 935.0386 | 701.5308 |
| MDGASNVTCcamINSR_M7 | 1483.0807 | 989.0562 | 742.0440 |
| MDGASNVTCcamINSR_M8 | 1564.1071 | 1043.0738 | 782.5572 |
| MDGASNVTCcamINSR_M9 | 1645.1335 | 1097.0914 | 823.0704 |
| MDGASNVTCcamINSR_MM | 1158.9751 | 772.9858 | 579.9912 |
| MDGASNVTCcamINSR_NaA_2P | 1735.6762 | 1157.4532 | 868.3417 |
| MDGASNVTCcamINSR_NaA_3P | 1801.6973 | 1201.4673 | 901.3523 |
| MDGASNVTCcamINSR_NaA_4P | 1867.7185 | 1245.4814 | 934.3629 |
| MDGASNVTCcamINSR_NaA_5P | 1933.7396 | 1289.4955 | 967.3735 |
| MDGASNVTCcamINSR_NaA_6P | 1999.7608 | 1333.5096 | 1000.3840 |
| MDGASNVTCcamINSR_NaA_P | 1669.6550 | 1113.4391 | 835.3312 |
| MDGASNVTCcamINSR_NaGn | 1588.6286 | 1059.4215 | 794.8179 |
| MDGASNVTCcamINSR_NaGn_P | 1654.6497 | 1103.4356 | 827.8285 |
| MDGASNVTCcamINSR_NaM | 1487.0889 | 991.7284 | 744.0481 |
| MDGASNVTCcamINSR_NaNa | 1815.2027 | 1210.4709 | 908.1050 |
| MDGASNVTCcamINSR_NaNa_P | 1947.2450 | 1298.4991 | 974.1261 |
| MoxDGASNVTCcamINSR_AA | 1532.1048 | 1021.7389 | 766.5560 |
| MoxDGASNVTCcamINSR_AA_2P | 1664.1471 | 1109.7671 | 832.5772 |
| MoxDGASNVTCcamINSR_AA_3P | 1730.1682 | 1153.7812 | 865.5878 |
| MoxDGASNVTCcamINSR_AA_4P | 1796.1894 | 1197.7953 | 898.5983 |
| MoxDGASNVTCcamINSR_AA_5P | 1862.2105 | 1241.8094 | 931.6089 |
| MoxDGASNVTCcamINSR_AA_6P | 1928.2317 | 1285.8235 | 964.6195 |
| MoxDGASNVTCcamINSR_AA_P | 1598.1259 | 1065.7530 | 799.5666 |
| MoxDGASNVTCcamINSR_AGn | 1451.0784 | 967.7213 | 726.0428 |
| MoxDGASNVTCcamINSR_AGn_2P | 1583.1207 | 1055.7495 | 792.0640 |
| MoxDGASNVTCcamINSR_AGn_3P | 1649.1418 | 1099.7636 | 825.0746 |
| MoxDGASNVTCcamINSR_AGn_4P | 1715.1630 | 1143.7777 | 858.0851 |
| MoxDGASNVTCcamINSR_AGn_5P | 1781.1841 | 1187.7918 | 891.0957 |
| MoxDGASNVTCcamINSR_AGn_6P | 1847.2053 | 1231.8059 | 924.1063 |
| MoxDGASNVTCcamINSR_AGn_P | 1517.0995 | 1011.7354 | 759.0534 |
| MoxDGASNVTCcamINSR_AM | 1349.5387 | 900.0282 | 675.2730 |
| MoxDGASNVTCcamINSR_AM_2P | 1481.5810 | 988.0564 | 741.2941 |
| MoxDGASNVTCcamINSR_AM_3P | 1547.6021 | 1032.0705 | 774.3047 |
| MoxDGASNVTCcamINSR_AM_4P | 1613.6233 | 1076.0846 | 807.3153 |
| MoxDGASNVTCcamINSR_AM_5P | 1679.6444 | 1120.0987 | 840.3259 |
| MoxDGASNVTCcamINSR_AM_6P | 1745.6656 | 1164.1128 | 873.3364 |
| MoxDGASNVTCcamINSR_AM_P | 1415.5598 | 944.0423 | 708.2836 |
| MoxDGASNVTCcamINSR_GnGn | 1370.0520 | 913.7037 | 685.5296 |
| MoxDGASNVTCcamINSR_GnM | 1268.5123 | 846.0106 | 634.7598 |
| MoxDGASNVTCcamINSR_GnM5 | 1430.5651 | 954.0458 | 715.7862 |
| MoxDGASNVTCcamINSR_M4 | 1247.9990 | 832.3351 | 624.5031 |
| MoxDGASNVTCcamINSR_M5 | 1329.0254 | 886.3527 | 665.0163 |
| MoxDGASNVTCcamINSR_M6 | 1410.0518 | 940.3703 | 705.5295 |
| MoxDGASNVTCcamINSR_M7 | 1491.0782 | 994.3879 | 746.0427 |
| MoxDGASNVTCcamINSR_M8 | 1572.1046 | 1048.4055 | 786.5559 |
| MoxDGASNVTCcamINSR_M9 | 1653.1310 | 1102.4231 | 827.0691 |
| MoxDGASNVTCcamINSR_MM | 1166.9726 | 778.3175 | 583.9899 |
| MoxDGASNVTCcamINSR_NaA_2P | 1743.6736 | 1162.7848 | 872.3405 |
| MoxDGASNVTCcamINSR_NaA_3P | 1809.6948 | 1206.7989 | 905.3510 |
| MoxDGASNVTCcamINSR_NaA_4P | 1875.7159 | 1250.8130 | 938.3616 |
| MoxDGASNVTCcamINSR_NaA_5P | 1941.7371 | 1294.8271 | 971.3722 |
| MoxDGASNVTCcamINSR_NaA_6P | 2007.7582 | 1338.8412 | 1004.3828 |
| MoxDGASNVTCcamINSR_NaA_P | 1677.6525 | 1118.7707 | 839.3299 |
| MoxDGASNVTCcamINSR_NaGn | 1596.6261 | 1064.7531 | 798.8167 |
| MoxDGASNVTCcamINSR_NaGn_P | 1662.6472 | 1108.7672 | 831.8272 |
| MoxDGASNVTCcamINSR_NaM | 1495.0864 | 997.0600 | 748.0468 |
| MoxDGASNVTCcamINSR_NaNa | 1823.2002 | 1215.8025 | 912.1037 |
| MoxDGASNVTCcamINSR_NaNa_P | 1955.2424 | 1303.8307 | 978.1249 |

**Supplementary Table 3.** Names and corresponding cartoons for all glycan structures described in this work. The cartoons are drawn based on the recommendations of the Consortium for Functional Glycomics (www.functionalglycomics.org).


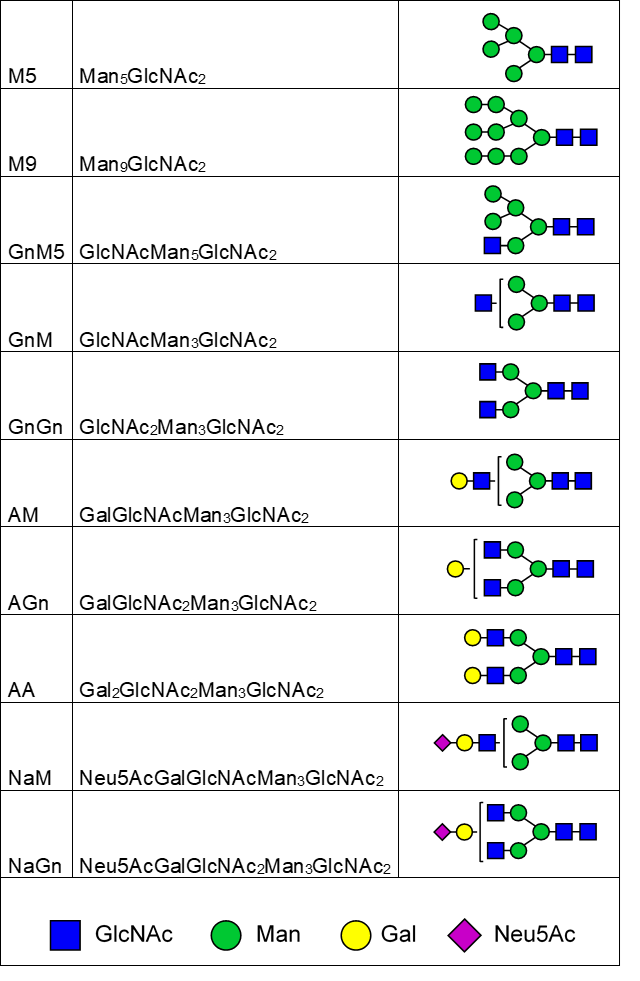


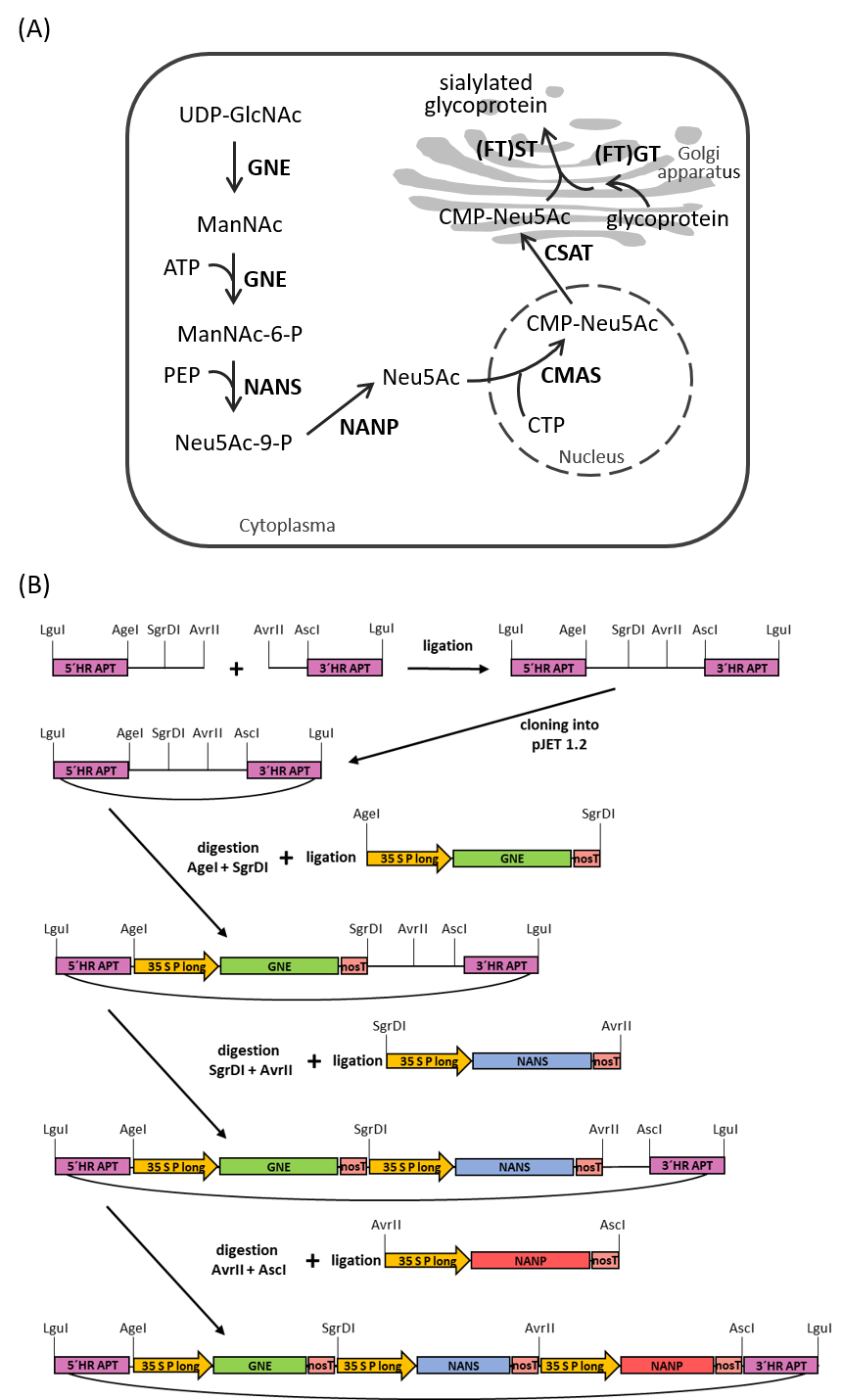


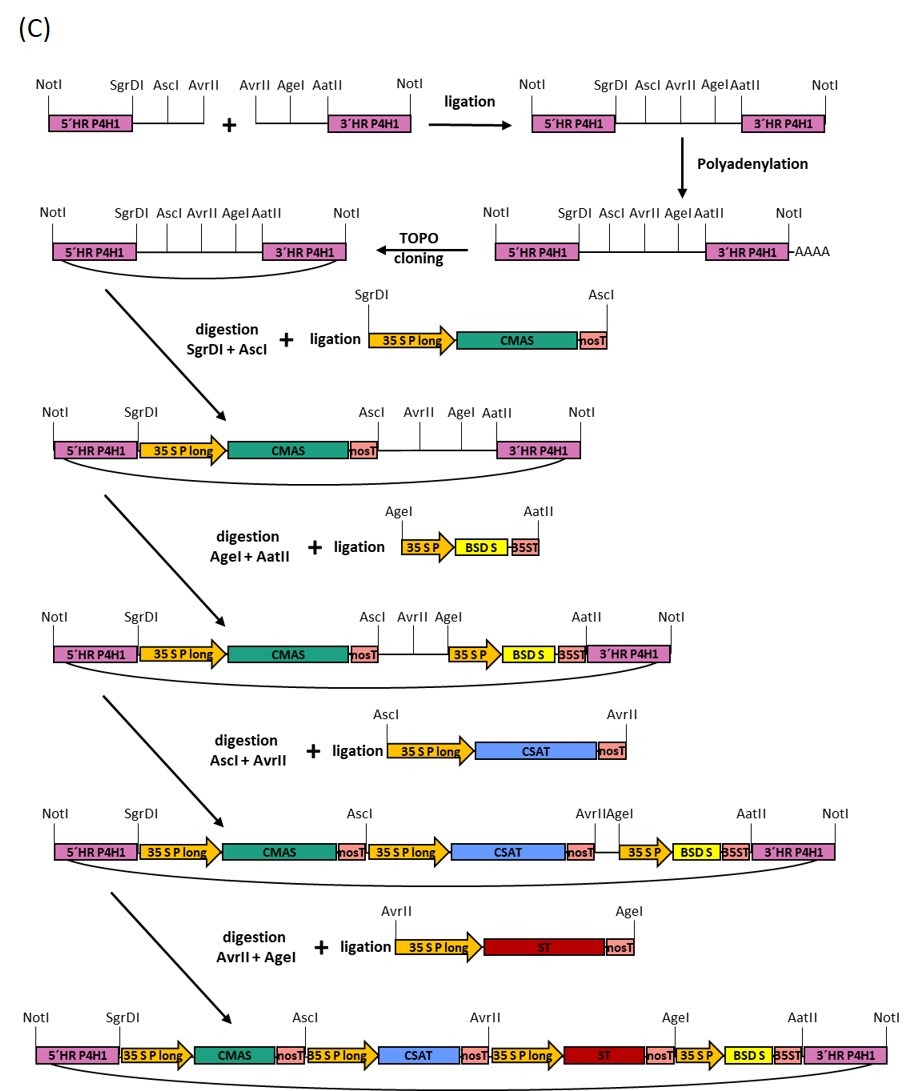


**Supplementary Figure 1.** Schematic representation of the sialic acid pathway in mammals and overview of the cloning procedure for the GNN and CCSB multi-gene constructs. **(A)** Diagram of the metabolic pathway for protein sialylation in mammals. **(B)** Successive cloning procedure of the GNN construct starting with the generation of the pJET1.2-based assembly vector with a designed multiple cloning site and homologous flanks for integration into the adenine phosphoribosyltransferase (APT, Pp3c8_16590V3.1) locus. This vector was subsequently linearized with two restriction enzymes cutting within the designed MCS and ligated with the respective transgene expression cassette digested with the same enzymes from the expression vector. This was performed subsequently for the GNE-, NANS, and NANP-expression cassettes, resulting in the assembled GNN construct. **(C)** Successive cloning procedure of the CCSB construct starting with the generation of the pTOPO-based assembly vector with a designed multiple cloning site and homologous flanks for integration into the prolyl-4-hydroxylase 1 (P4H1, Pp3c8_7140V3.1) locus. This vector was subsequently linearized with two restriction enzymes cutting within the designed MCS and ligated with the respective transgene expression cassette digested with the same enzymes from the expression vector. This was performed subsequently for the CMAS, CSAT, ST and BSD-expression cassettes, resulting in the assembled CCSB construct. The restriction enzymes used are indicated above the constructs. 5´HR: 5´-homologous region, 3´HR: 3´-homologous region, 35SP_long: long CaMV 35S promoter, 35SP: CaMV 35S promoter, nosT: nos terminator, 35ST: CaMV 35S terminator.

**
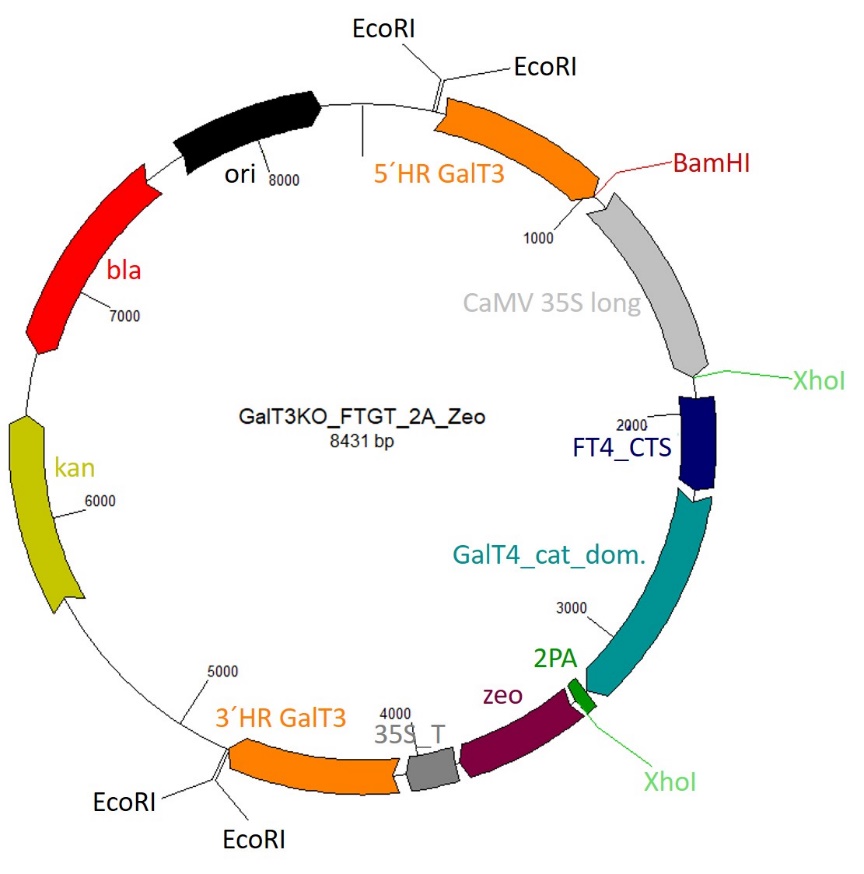
**

**Supplementary Figure 2.** Schematic illustration of the plasmid containing the FTGT expression construct for targeted integration into the GalT3 locus. The homologous 5´- and 3´-flanks (5´HR GalT3, 3´HR GalT3) for targeted integration into the β1,3-galactosyltransferase  1 (GalT3) locus are displayed in orange. CaMV 35S long: long CaMV 35S promoter, FT4_CTS: sequence encoding the N-terminus of moss α1,4‑fucosyltransferase including the CTS domain; GalT4_cat_dom.: sequence encoding the catalytic domain of the human GalT4, P2A: 2A peptide sequence from the porcine teschovirus-1, zeo: zeocine resistance coding for bleomycin resistance protein, 35S_T:CaMV 35S terminator, kan: kanamycin selection cassette, bla: ampicillin resistance cassette coding for beta-lactamase.


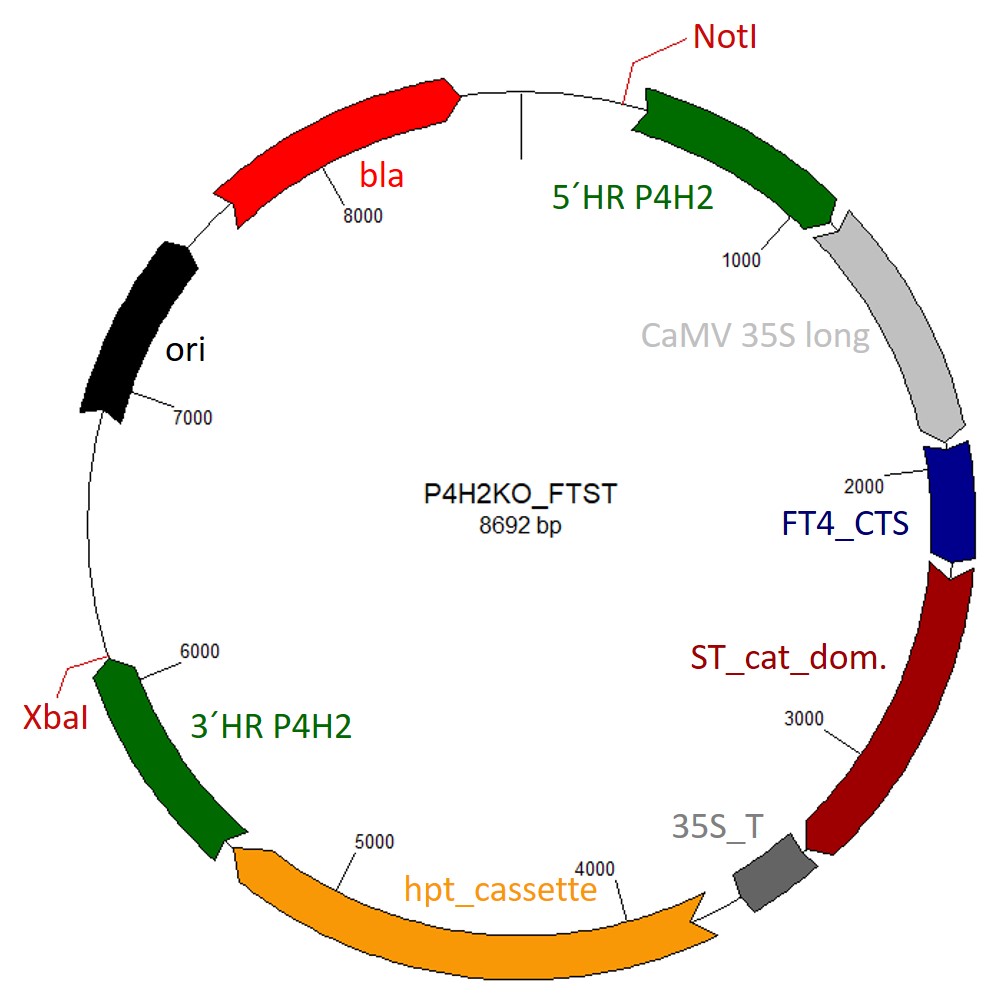


**Supplementary Figure 3.** Schematic illustration of the plasmid containing the FTST expression construct for targeted integration into the P4H2 locus. The homologous 5´- and 3´-flanks (5´HR_P4H2, 3´HR_P4H2) for targeted integration into the prolyl-4-hydroxylase 2 (P4H2) locus are displayed in green. CaMV 35S long: long CaMV 35S promoter, FT4_CTS: sequence encoding the N-terminus of moss α1,4‑fucosyltransferase including the CTS domain; ST_cat_dom.: sequence encoding the catalytic domain of the rat α2,6-sialyltransferase, 35S_T: CaMV 35S terminator, hpt_cassette: hygromycin B phosphotransferase encoding resistance gene driven by the nos promotor and nos terminator, bla: ampicillin resistance cassette coding for beta-lactamase.


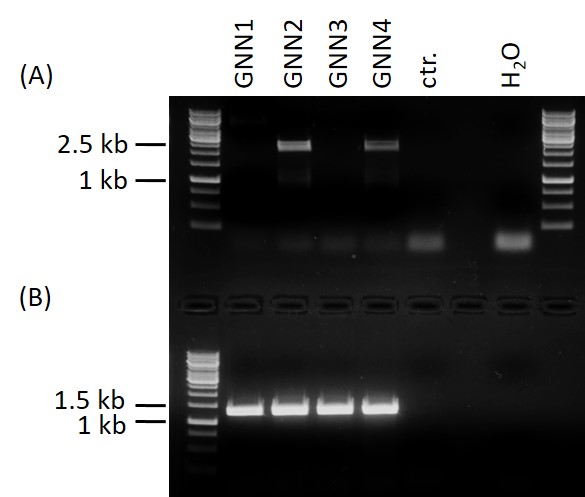


**Supplementary Figure 4.** PCR-based analysis of 5´- (A) and 3´-integration (B) of the GNN construct within the APT locus. PCR reactions were performed using primer pairs 53 and 54 for 5´- and 55 and 56 for 3´-integration on genomic DNA of lines GNN1-4 as well as of parental line as negative control (ctr.). To demonstrate the absence of contamination a control without DNA (H_2_O) was included. Amplicons of 2244 bp for the 5´- (**(A)**, GNN2 and GNN4) or 1311 bp for the 3´-integration **(B)** confirmed the targeted integration.


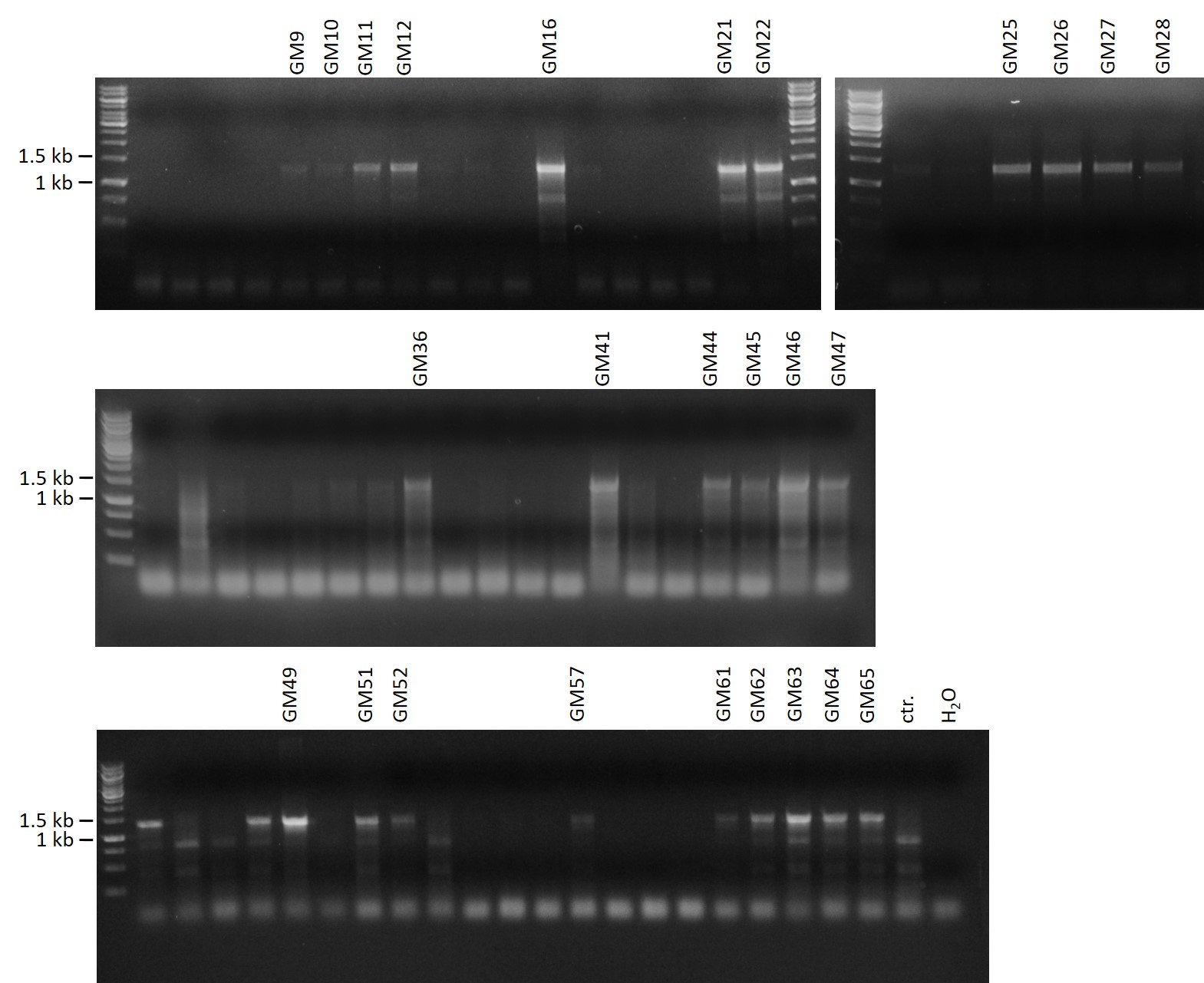


**Supplementary Figure 5.** PCR-based analysis of 3´-integration of the GM construct within the APT locus. PCR reactions were performed using the primer pairs 55 and 56 for 3´-integration on genomic DNA of lines GM1-65 as well as of a negative control (ctr.). To demonstrate the absence of contamination a control without DNA (H_2_O) was included. Amplicons of 1311 bp confirmed targeted 3´ construct integration.


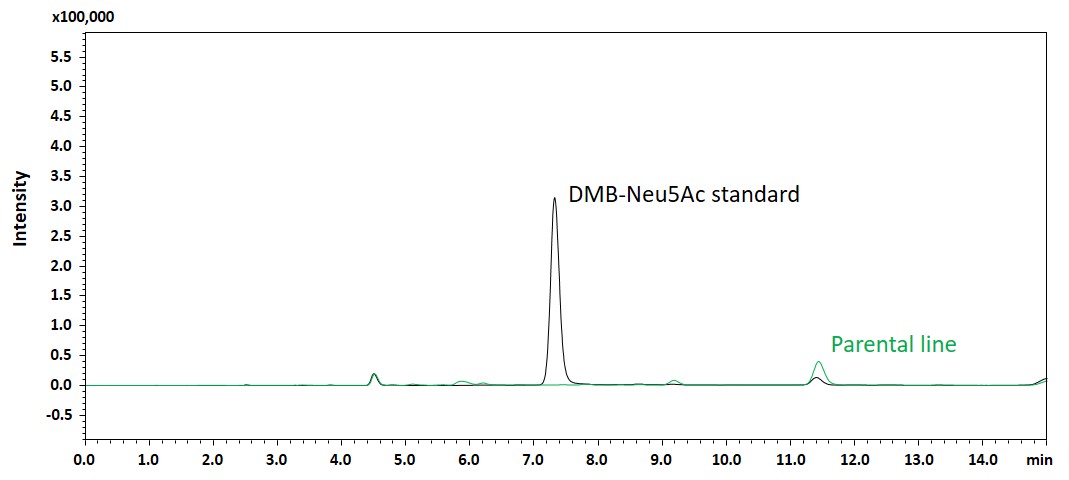


**Supplementary Figure 6.** Analysis for sialic acid in a DMB-labeled moss negative control in comparison to a DMB-Neu5Ac standard via reverse-phase high-performance liquid chromatography coupled with fluorescence detection (RP-HPLC-FLD). An extract of the parental plant of GNN and GM lines was treated with 1,2-diamino-4,5-methylenedioxybenzene (DMB) to derivatize Neu5Ac into DMB-Neu5Ac. No interfering products in the elution time for DMB-Neu5Ac could be detected in the moss negative control (green line) compared to the DMB-Neu5Ac standard (black line).


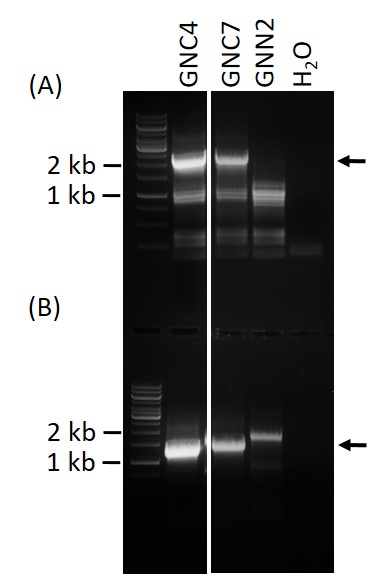


**Supplementary Figure 7.** PCR-based analysis of 5´- and 3´-integration of the CCSB construct in the P4H1 locus. PCR reactions were performed using primer pairs 57 and 58 for 5´- and 59 and 60 for 3´-integration on genomic DNA of lines GNC4 and GNC7 as well as of the GNN2-parental line as negative control. To demonstrate the absence of contamination a control without DNA (H_2_O) was included. Amplicons of 2222 bp for the 5´- **(A)** or 1406 bp for the 3´-integration **(B)** confirmed the targeted integration in the lines GNC4 and GNC7 (indicated by the black arrows).

**
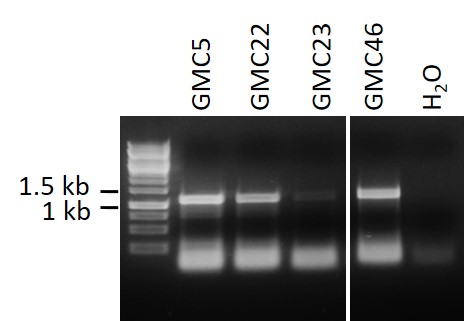
**

**Supplementary Figure 8.** PCR-based analysis of 3´-integration of the CCSB construct in the P4H1 locus. PCR reactions were performed using primer pair 59 and 60 for 3´-integration on genomic DNA of lines GMC5, 22, 23, and 46, resulting from CCSB transfections of line GM28. To demonstrate the absence of contamination a control without DNA (H_2_O) was included. Amplicons of 1406 bp confirmed targeted 3´ construct integration in all analyzed lines.


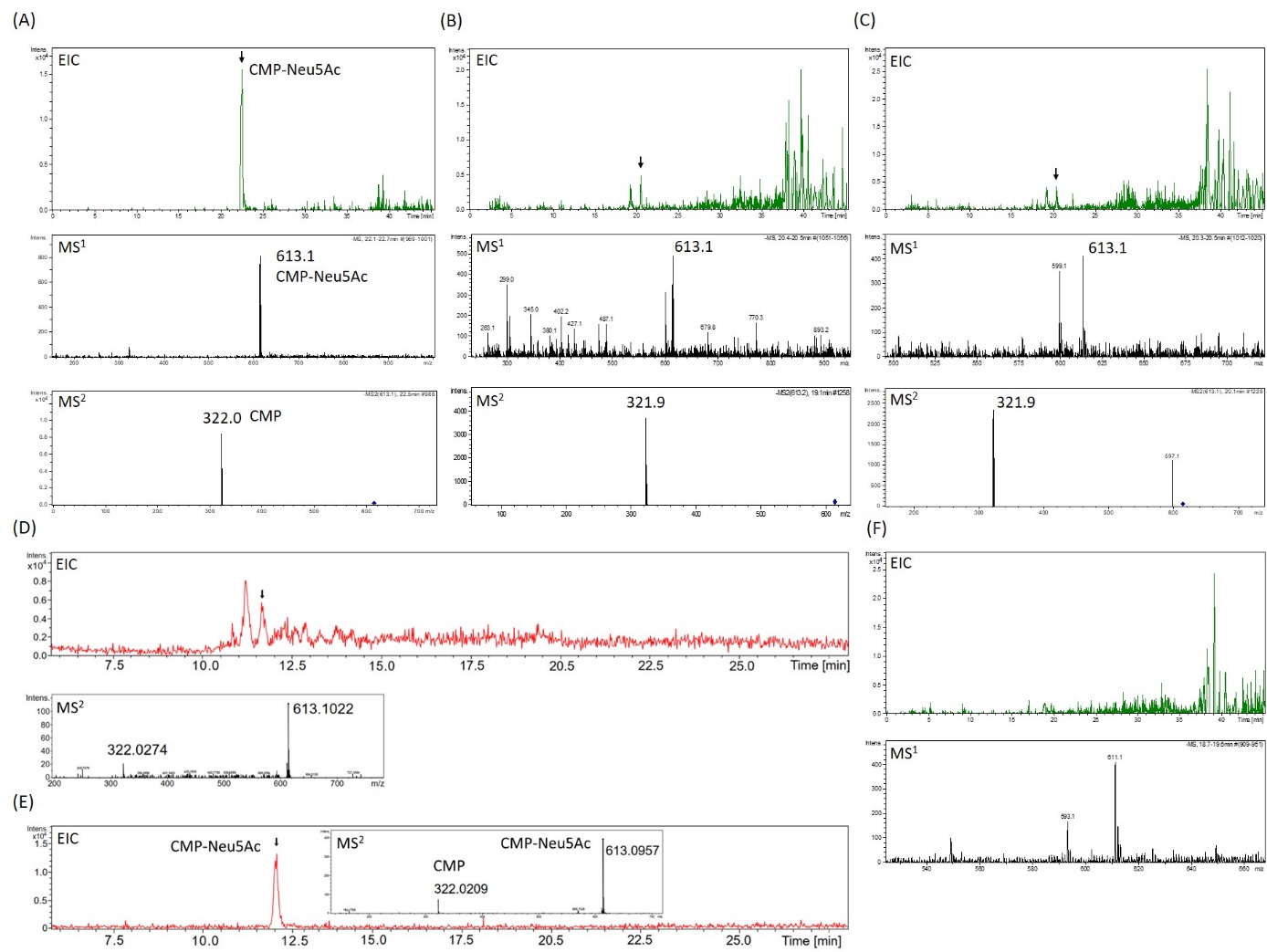


**Supplementary Figure 9.** Mass-spectrometric detection of CMP-Neu5Ac in moss extracts. Extracted ion chromatogram (*m/z* of 613.1 ± 0.1) of LC-MS of 70 pmol of a CMP-Neu5Ac standard **(A)** used as positive control for extracts of the lines GMC23 **(B)** and GMC46 **(C)**, line GNC7 **(D)** with its respective control of CMP-Neu5Ac standard **(E)**, and the sialic acid non-activating parental line as negative control **(F)**. In all measurements, expect for the negative control, peaks corresponding to the [M‑H]^−^-ion of CMP-Neu5Ac of *m/z* 613.1 could be detected on MS^1^ level (middle panels A-C, E and lower panel of D). Confirmation of corresponding MS^1^-peak identities (indicated by the black arrows) was performed on MS^2^ level via the identification of the [M-H]^‑^ CMP fragment ion of *m/z* 322.0 (lower panels A-D and E). CMP-Neu5Ac concentration of the moss extracts was determined via peak area integration in comparison to defined standard values and resulted in 14 nmol CMP-Neu5Ac/g DW in the lines GMC23 and 46 and 2.3 nmol CMP-Neu5Ac/g DW in GNC7.


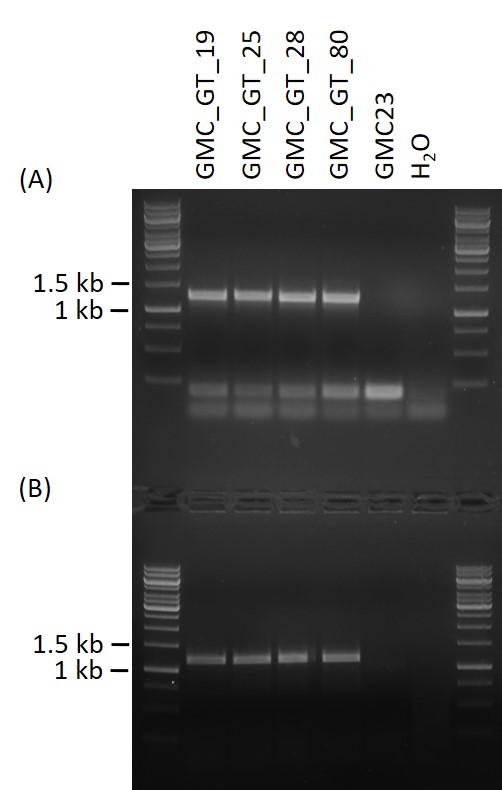


**Supplementary Figure 10.** PCR-based analysis of 5´-and 3´-integration of the FTGT construct in the GalT3 locus. PCR reactions were performed on genomic DNA of the lines GMC_GT_19, 25, 28 and 80 as well as the GMC23 parental line using the primer pairs 61 and 62 for 5´- as well as 63 and 64 for 3´-integration, respectively. To demonstrate the absence of contamination a control without DNA (H_2_O) was included. These analyses resulted in integration-confirming amplicons of 1253 for the 5´- **(A)** and 11171 bp for the 3´-integration **(B)** for all transgenic lines, whereas no integration-indicating signals were obtained in the parental line and the H_2_O control.


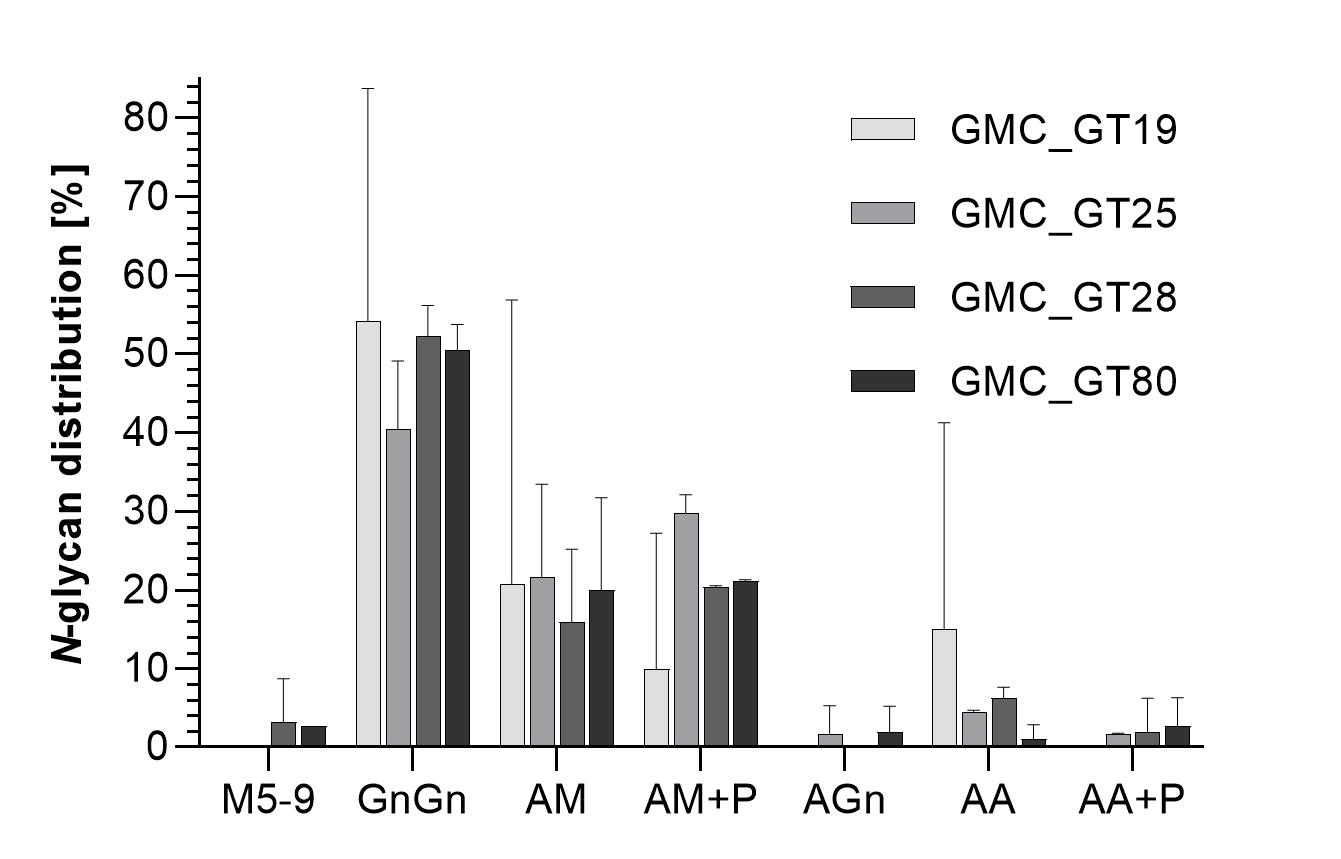


**Supplementary Figure 11.** Mass-spectrometric analysis of *N*-glycosylation patterns in GMC_GT lines. MS-based relative quantification of identified tryptic glycopeptides from the reporter glycoprotein of complete sialyation lines. Relative quantification was based on peak area integration of extracted ion chromatograms on MS^1^ level, for which peak identities were confirmed on MS^2^ level. For quantification, areas of all confirmed peaks per measurement were summed up and the relative percentages are given for each identified glycan structure. The error bars indicate the standard deviation between the two detected glycopeptides. M: mannose, Gn: *N*-acetylglucosamine, A: galactose, P: represents the presence of one or two pentoses attached to the *N*-glycan.


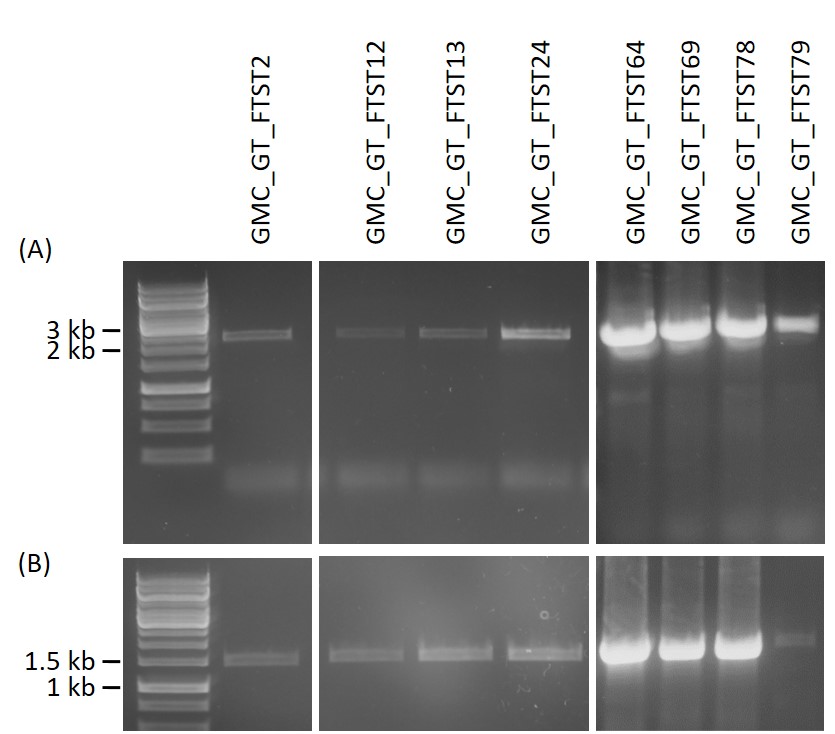


**Supplementary Figure 12.** PCR signals confirming targeted 5´-and 3´-integration of the FTST construct within the P4H2 locus. PCR reactions were performed on genomic DNA using the primer pairs 65 and 66 for 5´- as well as 67 and 68 for 3´- integration, respectively. These analyses resulted for all analyzed lines in integration-confirming amplicons of 2169 for the 5´- **(A)** and 1464 bp for the 3´-integration **(B)**, respectively.


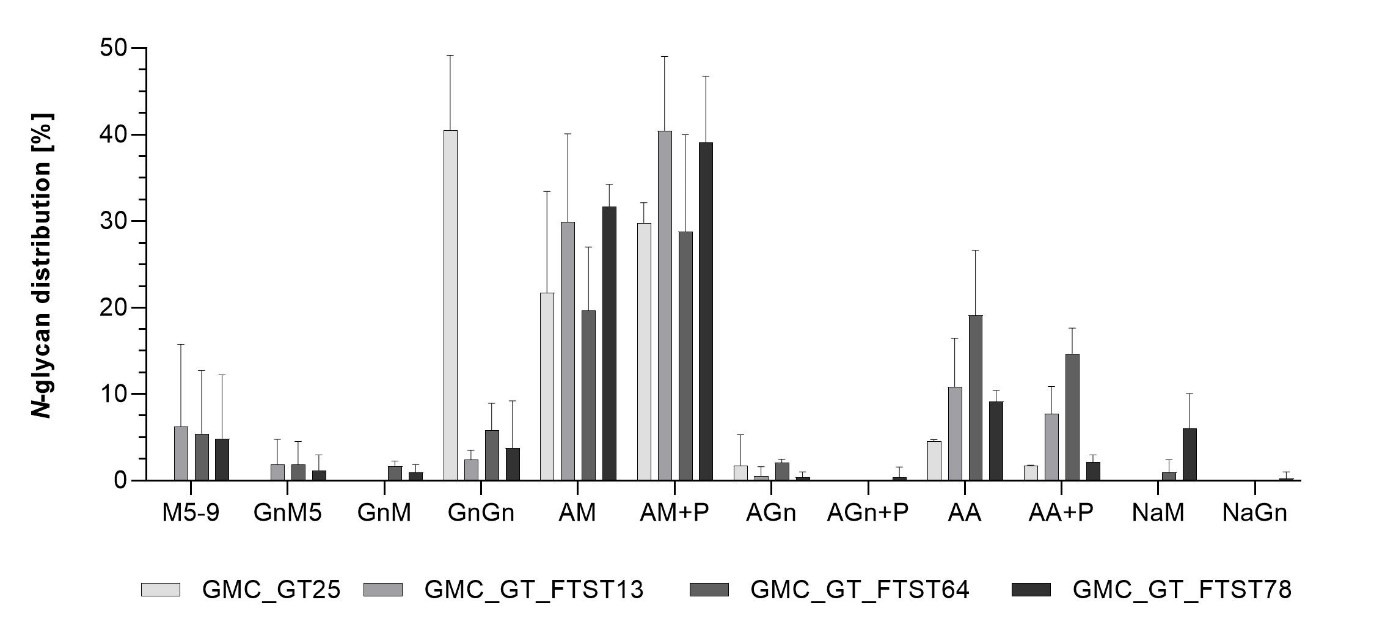


**Supplementary Figure 13.** Mass-spectrometric analysis of glycosylation patterns in FTST-expressing lines compared to the GMC_GT25 parental line. MS-based relative quantification of glycopeptides identified on the tryptically digested reporter glycoprotein. Relative quantification was based on peak area integration of extracted ion chromatograms on MS^1^ level, for which peak identities were confirmed on MS^2^ level. For quantification, areas of all confirmed peaks per measurement were summed up and the relative percentages are given for each identified glycan structure. The error bars indicate the standard deviation between the three analyzed glycopeptides. M: mannose, Gn: *N-*acetylglucosamine, A: galactose, Na: sialic acid, P: indicates the presence of one or two pentoses on the corresponding *N-*glycan structure.


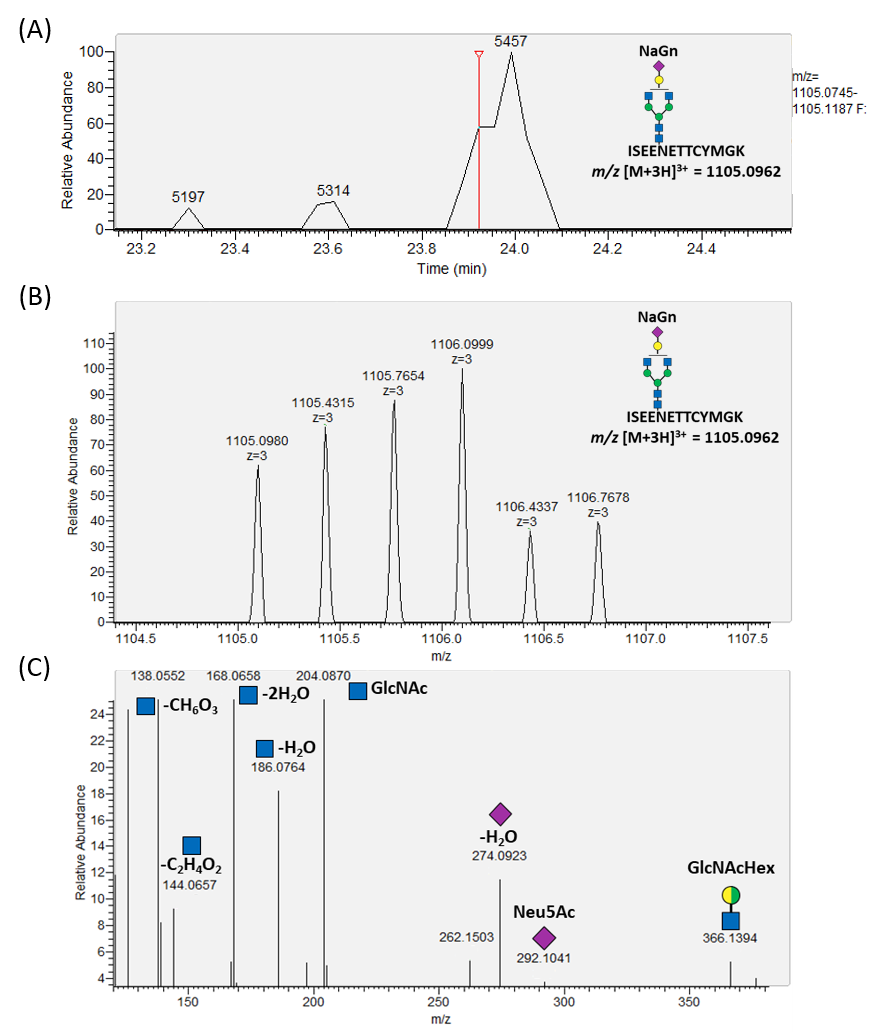
**Supplementary Figure 14.** Mass-spectrometric verification of the identified NaGn-glycosylation of the ISEENETTCYMGK glycopeptide on MS^1^ **(A+B)** and MS^2^ level **(C)**. **(A)** Extracted ion chromatogram of the expected [M+3H]^3+^ m/z-ratio of 1105.0962 of the tryptic NaGn-ISEENETTCYMGK glycopeptide on MS^1^ level. **(B)** Verification of the charge state (z=3) of the detected glycopeptide via investigation from the isotope pattern. **(C)** MS^2^-based verification of N‑glycan sialylation via the detection of *N*‑acetylglucosamine (GlcNAc) and sialic acid (Neu5Ac) reporter ions with the following m/z-values: [GlcNAc]^+^ = 204.087, [GlcNAc - H_2_O]^+^ = 186.076, [GlcNAc - 2H_2_O]^+^ = 168.066, [GlcNAc - C_2_H_4_O_2_]^+^ = 144.065, [GlcNAc - CH_6_O_3_]^+^ = 138.055, [GlcNAc - C_2_H_6_O_3_]^+^ = 126.055), [Neu5Ac]^+^ = 292.103, [Neu5Ac - H_2_O]^+^ = 274.092 and the detection of the glycan fragment ion [GlcNAcHex]^+^ = 366.139. Blue square: *N*‑acetylglucosamine (Gn, GlcNAc), green circle: mannose (M), yellow circle: galactose (A), purple rhombus: sialic acid (Na, Neu5Ac), Hex: hexose (yellow and green circle: stands for the presence of either mannose or galactose).


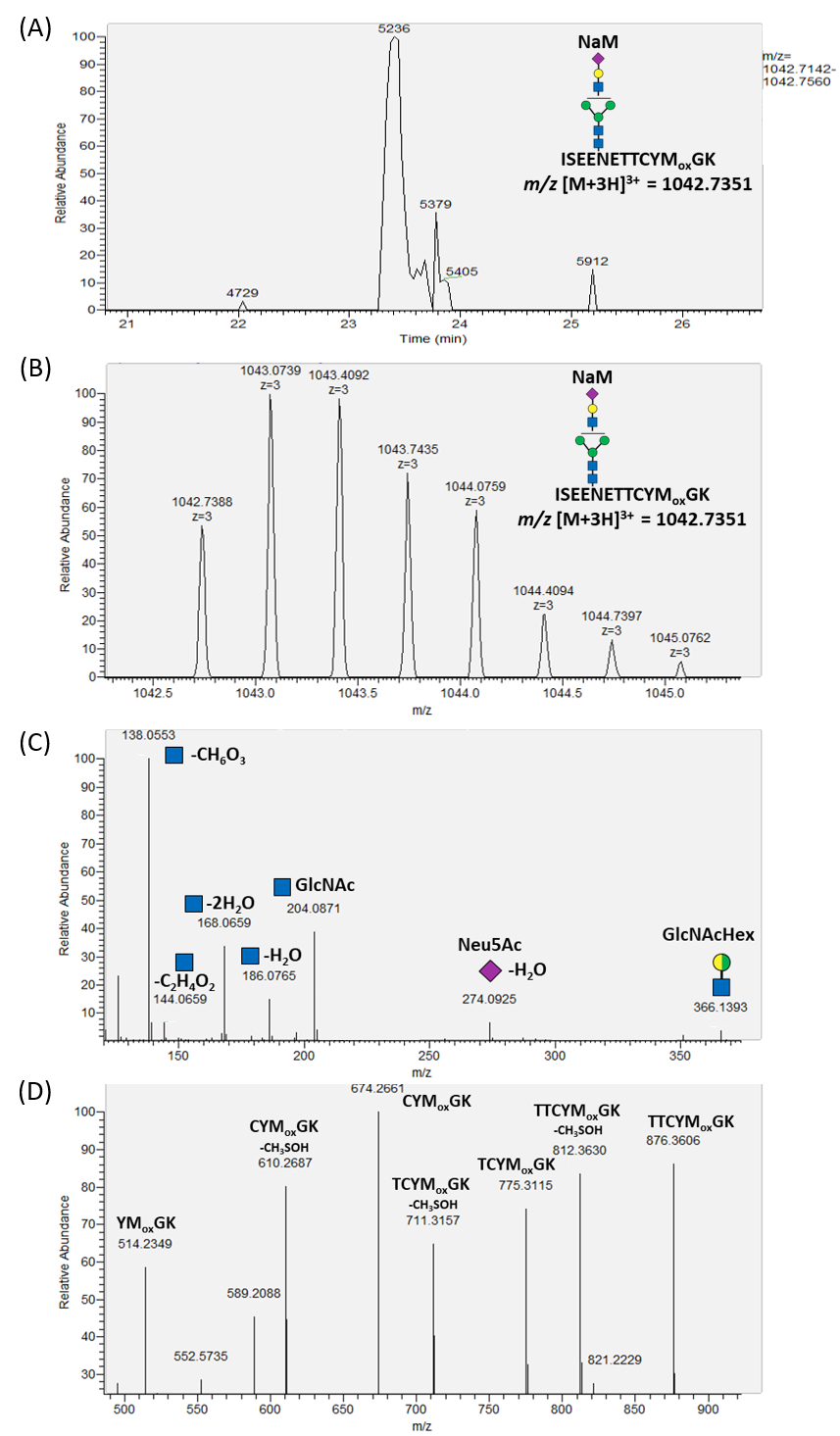


**Supplementary Figure 15.** Mass spectrometric verification of the identified NaM-glycosylation of the ISEENETTCYM_ox_GK glycopeptide on MS^1^ **(A+B)** and MS^2^ level **(C+D)**. **(A)** Extracted ion chromatogram of the expected [M+3H]^3+^ m/z-ratio of 1042.7351 of the tryptic NaM-ISEENETTCYM_ox_GK glycopeptide on MS^1^ level. **(B)** Verification of the charge state (z=3) of the detected glycopeptide via investigation from the isotope pattern. **(C)** MS^2^-based verification of N‑glycan sialylation via the detection of N‑acetylglucosamine (GlcNAc) and sialic acid (Neu5Ac) reporter ions with the following m/z-values: [GlcNAc]^+^ = 204.087, [GlcNAc - H_2_O]^+^ = 186.076, [GlcNAc - 2H_2_O]^+^ = 168.066, [GlcNAc - C_2_H_4_O_2_]^+^ = 144.065, [GlcNAc - CH_6_O_3_]^+^ = 138.055, [GlcNAc - C_2_H_6_O_3_]^+^ = 126.055), [Neu5Ac]^+^ = 292.103, [Neu5Ac - H_2_O]^+^ = 274.092 and the detection of the glycan fragment ion [GlcNAcHex]^+^ = 366.139. **(D)** Fragmentation of the peptide backbone. Blue square: N‑acetylglucosamine (Gn, GlcNAc), green circle: mannose (M), yellow circle: galactose (A), purple rhombus: sialic acid (Na, Neu5Ac), Hex: hexose (yellow and green circle: stands for the presence of either mannose or galactose).


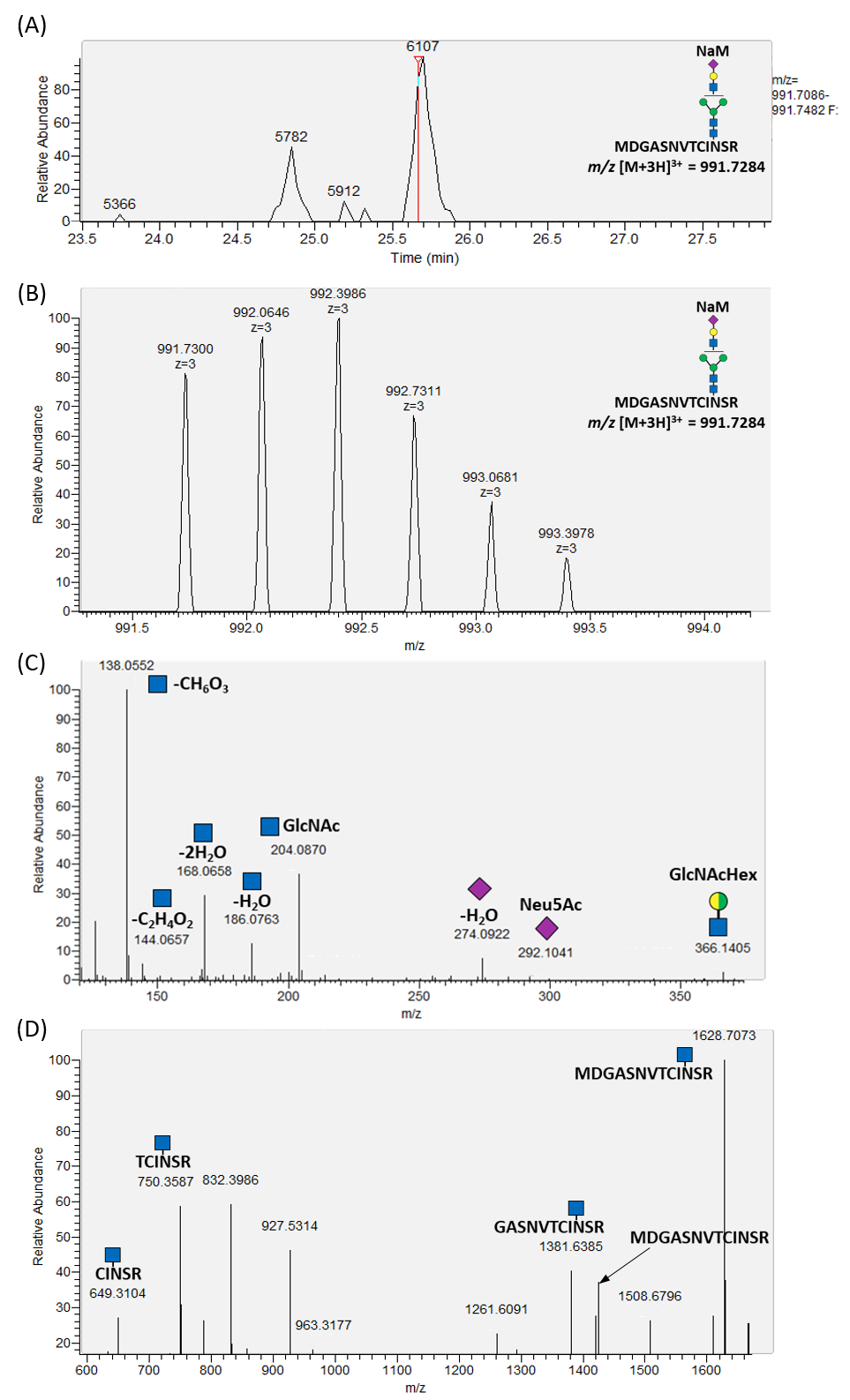


**Supplementary Figure 16.** Mass-spectrometric verification of the identified NaM-glycosylation of the MDGASNVTCINSR glycopeptide on MS^1^ **(A+B)** and MS^2^ level **(C+D). (A)** Extracted ion chromatogram of the expected [M+3H]^3+^ *m/z*-ratio of 991.7284 of the tryptic NaM-MDGASNVTCINSR glycopeptide on MS^1^ level. **(B)** Verification of the charge state (z=3) of the detected glycopeptide via investigation from the isotope pattern. **(C)** MS^2^-based verification of *N*‑glycan sialylation via the detection of *N‑*acetylglucosamine (GlcNAc) and sialic acid (Neu5Ac) reporter ions with the following *m/z*-values: [GlcNAc]^+^ = 204.087, [GlcNAc - H_2_O]^+^ = 186.076, [GlcNAc - 2H_2_O]^+^ = 168.066, [GlcNAc - C_2_H_4_O_2_]^+^ = 144.065, [GlcNAc - CH_6_O_3_]^+^ = 138.055, [GlcNAc - C_2_H_6_O_3_]^+^ = 126.055), [Neu5Ac]^+^ = 292.103, [Neu5Ac - H_2_O]^+^ = 274.092 and the detection of the glycan fragment ion [GlcNAcHex]^+^ = 366.139. **(D)** Fragment ions of the glycopeptide. Blue square: *N‑*acetylglucosamine (Gn, GlcNAc), green circle: mannose (M), yellow circle: galactose (A), purple rhombus: sialic acid (Na*,* Neu5Ac), Hex: hexose (yellow and green circle: stands for the presence of either mannose or galactose).


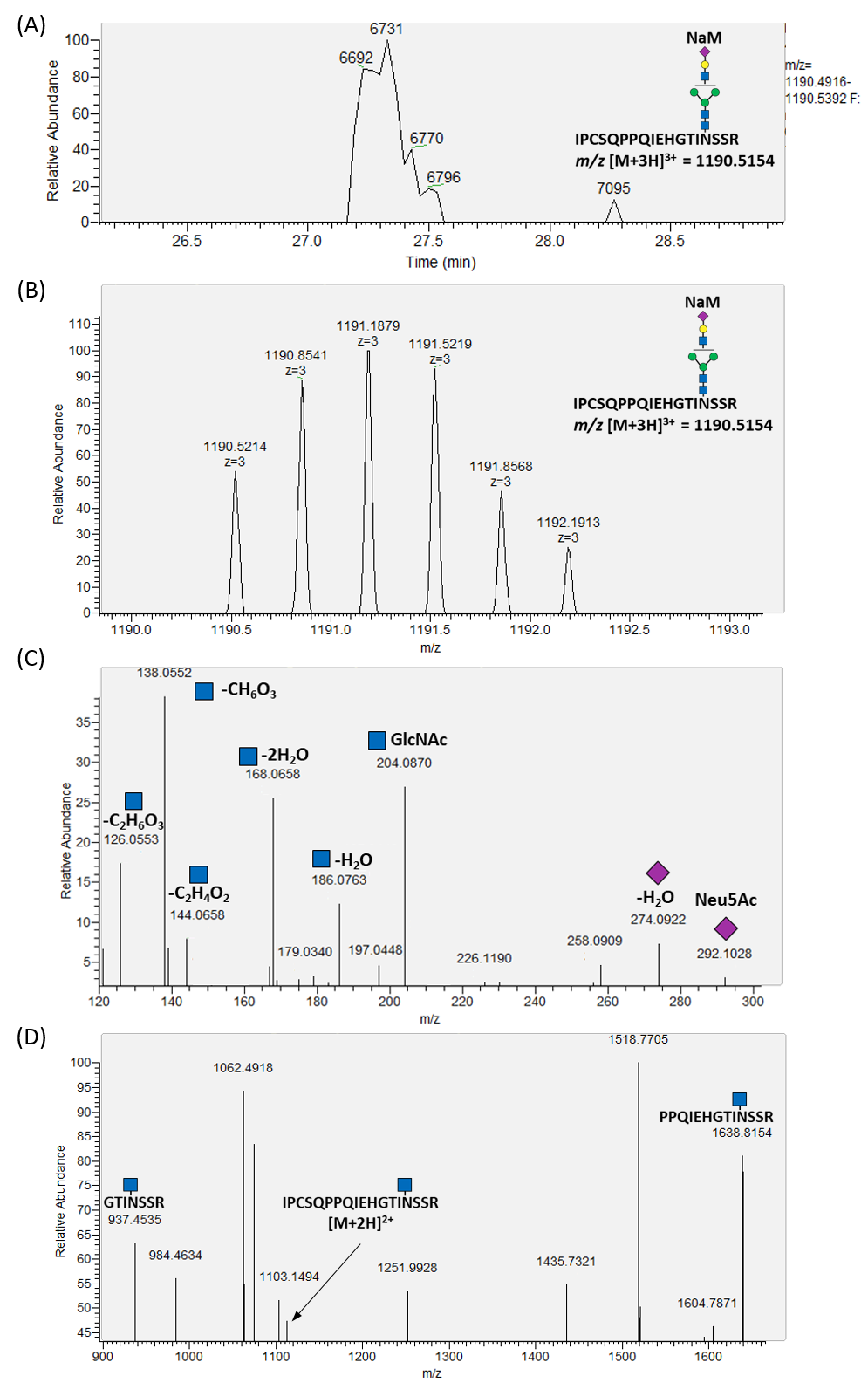


**Supplementary Figure 17.** Mass-spectrometric verification of the identified NaM-glycosylation of the IPCSQPPQIEHGTINSSR glycopeptide on MS^1^ **(A+B)** and MS^2^ level **(C+D).** **(A)** Extracted ion chromatogram of the expected [M+3H]^3+^ *m/z*-ratio of 1090.5154 of the tryptic MNa-IPCSQPPQIEHGTINSSR glycopeptide on MS^1^ level. **(B)** Verification of the charge state (z=3) of the detected glycopeptide via investigation from the isotope pattern. **(C)** MS^2^-based verification of *N*‑glycan sialylation via the detection of *N‑*acetylglucosamine (GlcNAc) and sialic acid (Neu5Ac) reporter ions with the following *m/z*-values: [GlcNAc]^+^ = 204.087, [GlcNAc - H_2_O]^+^ = 186.076, [GlcNAc - 2H_2_O]^+^ = 168.066, [GlcNAc - C_2_H_4_O_2_]^+^ = 144.065, [GlcNAc - CH_6_O_3_]^+^ = 138.055, [GlcNAc - C_2_H_6_O_3_]^+^ = 126.055), [Neu5Ac]^+^ = 292.103 and [Neu5Ac - H_2_O]^+^ = 274.092. **(D)** Fragment ions of the glycopeptide. Blue square: *N‑*acetylglucosamine (Gn, GlcNAc), green circle: mannose (M), yellow circle: galactose (A), purple rhombus: sialic acid (Na, Neu5Ac), Hex: hexose (yellow and green circle: stands for the presence of either mannose or galactose).


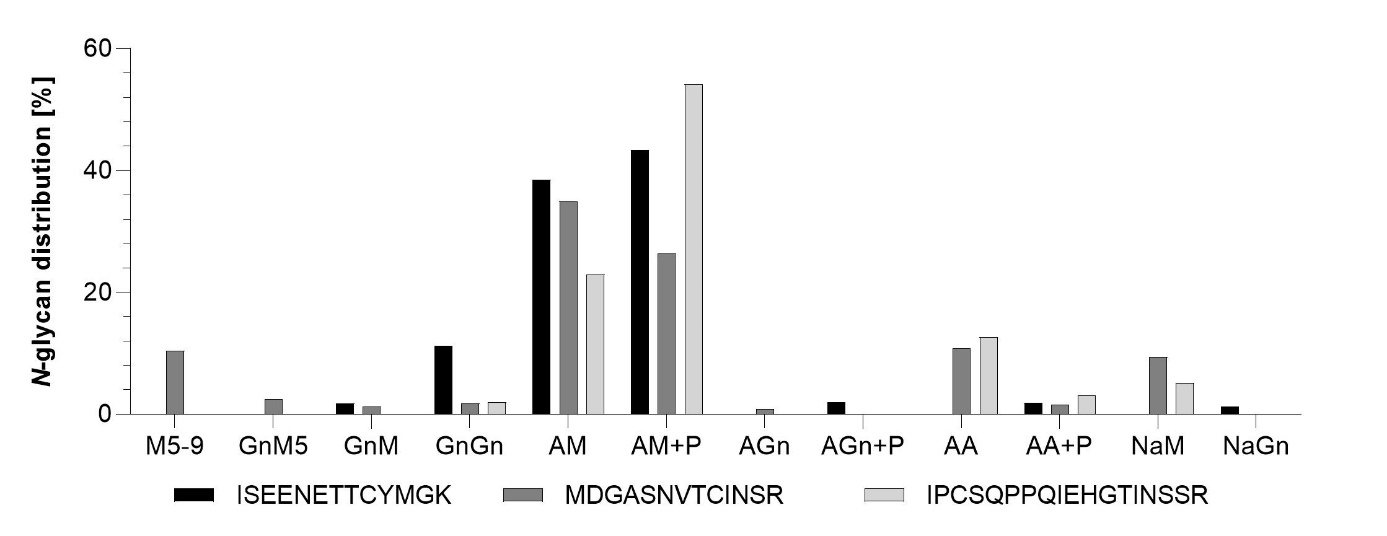


**Supplementary Figure 18.** Mass-spectrometric analysis of the *N*-glycosylation pattern at the analyzed tryptic glycopeptides ISEE**N**ETTCYMGK, MDGAS**N**VTCINSR and IPCSQPPQIEHGTI**N**SSR of the recombinant reporter protein produced in line GMC_GT_FTST78. MS-based relative quantification of *N*-glycan structures identified on the corresponding tryptic glycopeptide was based on peak area integration of extracted ion chromatograms (EICs) on MS^1^ level, for which glycopeptide identities were confirmed on MS^2^ level. For quantification, areas of all confirmed peaks per glycopeptide were summed up and the relative percentages are given for each identified glycan structure. M: mannose, Gn: *N*-acetylglucosamine, A: galactose, Na: sialic acid, P: indicates the presence of one or two pentoses on the corresponding *N*-glycan structure.
